# Prognostic value of nutritional and inflammation-based scores in advanced soft tissue sarcoma treated with trabectedin: a multicenter study

**DOI:** 10.3389/fonc.2026.1820227

**Published:** 2026-04-23

**Authors:** Seda Jeral Evinç, Çağla Eyüpler Akmercan, Selin Cebeci, Zeliha Birsin, İsmail Nazlı, Ali Kaan Güren, Selver Işık, Bahadır Köylü, İlknur Deliktaş Onur, Tugay Atasever, Tuğba Kaya, Emir Çerme, Murat Günaltılı, Berrin Papila, Nebi Serkan Demirci, Çiğdem Papila, Özkan Alan

**Affiliations:** 1 Department of Medical Oncology, Cerrahpaşa Faculty of Medicine, Istanbul University-Cerrahpaşa, Istanbul, Türkiye; 2 Department of Medical Oncology, Göztepe Prof. Dr. Süleyman Yalçın City Hospital, Istanbul, Türkiye; 3 Department of Medical Oncology, Pendik Training and Research Hospital, Istanbul, Türkiye; 4 Department of Medical Oncology, Koç University Faculty of Medicine, Istanbul, Türkiye; 5 Department of Medical Oncology, Dr. Abdurrahman Yurtaslan Oncology Training and Research Hospital, Ankara, Türkiye; 6 Department of Medical Oncology, Prof. Dr. Cemil Taşcıoğlu City Hospital, Istanbul, Türkiye; 7 Department of Medical Oncology, Dr. Lütfi Kırdar City Hospital, Istanbul, Türkiye; 8 Department of General Surgery, Cerrahpaşa Faculty of Medicine, Istanbul University-Cerrahpaşa, Istanbul, Türkiye

**Keywords:** inflammatory markers, modified Glasgow prognostic score, nutrition, overall survival, prognosis, soft tissue sarcoma, trabectedin

## Abstract

**Background:**

Trabectedin is a standard treatment for advanced soft tissue sarcomas (STS) after anthracycline failure. However, reliable prognostic markers remain limited. This study aims to evaluate the prognostic significance of inflammatory indices and the modified Glasgow Prognostic Score (mGPS) in STS patients treated with trabectedin.

**Methods:**

We retrospectively analyzed 60 patients with advanced soft tissue sarcoma treated with trabectedin between 2015 and 2024 across multiple tertiary oncology centers. Baseline laboratory parameters obtained within two days prior to treatment initiation were used to calculate neutrophil-to-lymphocyte ratio (NLR), platelet-to-lymphocyte ratio (PLR), pan-immune-inflammation value (PIV), inflammatory burden index (IBI), prognostic nutritional index (PNI), and modified Glasgow Prognostic Score (mGPS). Associations with overall survival were evaluated using Cox proportional hazards regression. Skewed continuous variables were log-transformed prior to analysis. To reduce the risk of overfitting, a limited number of clinically relevant variables were included in the multivariate model, while potential collinearity among inflammatory indices was considered.

**Results:**

A total of 37 patients (61.7%) were female, with a median age of 54.5 years (range: 35–77). Leiomyosarcoma was the predominant histological subtype, observed in 55% of cases. Trabectedin was administered at a median of the third-line setting (range: 2nd to 5th line). The objective response rate (ORR) was 24%, while the disease control rate (DCR) was 56%. The median follow-up duration was 10.2 months (range: 1–50.7). Median progression-free survival (PFS) was 4.9 months, and median overall survival (OS) was 11.7 months. Patients with mGPS 0 had significantly longer OS than those with mGPS 1 or 2 (median OS: 20.5 vs. 10.1 and 10.9 months; p = 0.006). In multivariate analysis, higher mGPS (1–2 vs 0) remained independently associated with worse OS (HR 3.9; 95% CI 1.5–10.0; p = 0.004).

**Conclusion:**

In this multicenter real-world cohort, mGPS was identified as an independent prognostic factor for overall survival in patients with advanced soft tissue sarcoma treated with trabectedin. These findings support its potential utility for prognostic stratification, warranting further prospective validation.

## 1 Introduction

Soft tissue sarcomas (STS) are rare malignancies of mesenchymal origin, accounting for approximately 1% of all adult cancers (1). According to the World Health Organization (WHO), they comprise close to 80 distinct histological subtypes, characterized by unique morphological, immunohistochemical, and molecular profiles. The broad histological spectrum of STS—from relatively common forms such as leiomyosarcoma and liposarcoma to uncommon entities like synovial sarcoma—makes prognostic evaluation challenging and limits the development of standardized therapeutic strategies (2). Although advances in surgery, radiotherapy, and systemic therapies have been made, the prognosis for patients with advanced or metastatic STS remains poor, with median overall survival in randomized first-line trials reported at approximately 12–14 months (3).

Trabectedin, an alkylating agent, is an established treatment option for patients with STS who progress after anthracycline-based chemotherapy (4). Its efficacy is particularly notable in leiomyosarcoma and liposarcoma, and beyond direct cytotoxicity, it also exerts immunomodulatory effects through interactions with monocytes and tumor-associated macrophages (5, 6). These properties may contribute to durable disease control in selected patients, though predictive factors for response remain to be defined.

Given the pronounced clinical and biological heterogeneity of STS, there is a clear need for reliable prognostic and predictive biomarkers. Systemic inflammatory indices such as the neutrophil-to-lymphocyte ratio (NLR) and the platelet-to-lymphocyte ratio (PLR) have been frequently investigated, but their prognostic significance has remained inconsistent, largely due to heterogeneous patient populations and the use of variable cut-off thresholds across studies which limit reproducibility and comparability of results (7–9). In recent years, composite indices that combine markers of systemic inflammation with nutritional status, such as the modified Glasgow Prognostic Score (mGPS), have attracted attention as potentially more reliable prognostic markers. Unlike NLR or PLR, which lack uniform cut-off values, mGPS relies on objective parameters—C-reactive protein and albumin—with predefined thresholds, and its prognostic role has been validated in cancers including non-small cell lung, gastrointestinal and hepatobiliary tumors (10–12). Thus, the aim of the present study was to evaluate the prognostic value of systemic inflammatory markers in a cohort of patients with advanced STS treated with trabectedin.

## 2 Materials and methods

### 2.1 Patients and methods

#### 2.1.1 Patients

We retrospectively collected demographic, clinicopathologic, and laboratory data from patients with soft tissue sarcoma who received trabectedin as part of a multicenter study conducted in Türkiye between 2015 and 2024. Inclusion criteria were: (1) histologically or cytologically confirmed diagnosis of STS; (2) documented disease progression following first-line therapy; (3) treatment with single-agent trabectedin, with at least one cycle administered; and (4) availability of complete medical records. Exclusion criteria were diagnosis of bone sarcoma, active infection, autoimmune disease, concurrent malignancy, age <18 years, use of systemic steroids within the last 3 days before baseline blood sampling, and insufficient clinical documentation. In addition, patients with conditions affecting albumin synthesis or protein loss, such as liver cirrhosis or renal disease associated with proteinuria, were also excluded. Baseline demographic, clinical, and laboratory data were recorded at the time of trabectedin initiation.

Ethics approval: This research has been approved by the authors’ affiliated institutions.

Informed consent: Informed consent was waived due to the retrospective nature of the study.

#### 2.1.2 Nutritional and Inflammation-Based Indices

Nutritional and inflammation-based indices were calculated using routine laboratory parameters, including neutrophil, lymphocyte, and platelet counts, as well as serum albumin and C-reactive protein (CRP) levels. All blood samples were obtained from routine blood tests performed within two days prior to the first dose of trabectedin.

Prognostic Nutritional Index (PNI) was calculated according to the method originally proposed by Onodera et al. (13) as follows:


$$PNI:[(10\times serum\;albumin(g/dL))+(0.005\times total\;lymphocyte\;count\;(10^{3}/\mu L))]$$

The Neutrophil-to-Lymphocyte Ratio (NLR) was defined as the absolute neutrophil count divided by the lymphocyte count, and the Platelet-to-Lymphocyte Ratio (PLR) was calculated as the platelet count divided by the lymphocyte count, as previously described in the literature (7).

The Inflammatory Burden Index (IBI) was determined by multiplying the CRP level (mg/L) by the neutrophil-to-lymphocyte ratio, in accordance with the original description of the index (14).

The Pan-Immune-Inflammation Value (PIV) was calculated using the following formula, as described in the literature (15):


PIV=(neutrophil count×platelet count×monocyte count)/lymphocyte count

The modified Glasgow Prognostic Score (mGPS) was calculated based on serum C-reactive protein (CRP) and albumin levels obtained prior to the initiation of trabectedin treatment (11). The scoring criteria were as follows:

mGPS-0: CRP <10 mg/L.
mGPS-1: CRP ≥10 mg/L and albumin ≥3.5 g/dL.
mGPS-2: CRP ≥10 mg/L and albumin <3.5 g/dL.

The mGPS was calculated in 54 patients; it could not be determined in 6 patients due to missing CRP and/or albumin data.

#### 2.1.3 Efficacy and safety outcomes

Progression-free survival (PFS) was defined as the time from the date of trabectedin initiation to the first radiologically or pathologically confirmed recurrence of disease, or death from any cause, whichever occurred first. Overall survival time (OS) was defined as the time from date of trabectedin initiation until death with any reason or the last documented clinical follow-up. Survival analysis was performed with Kaplan–Meier method. Patients who were lost to follow-up were censored at the time of their last documented contact.

Radiological tumor assessment was performed at baseline and every 8–12 weeks according to institutional practice and evaluated using the Response Evaluation Criteria in Solid Tumors (RECIST), version 1.1. Objective response rate (ORR) was defined as the proportion of patients who achieved complete or partial response, and disease control rate (DCR) as the proportion of patients who achieved complete response, partial response, or stable disease.

### 2.2 Statistical analysis

All statistical analyses were performed using IBM SPSS Statistics for Windows, version 25.0 (IBM Corp., Armonk, NY, USA). The normality of continuous variables was assessed using the Shapiro–Wilk or Kolmogorov–Smirnov tests, as appropriate. Continuous variables were expressed as median (range), and categorical variables as number (percentage). Comparisons between groups were performed using the independent samples t-test or the Mann–Whitney U test for continuous variables, and the chi-squared test or Fisher’s exact test for categorical variables. The prognostic value of inflammatory and nutritional indices was primarily assessed using Cox proportional hazards regression analysis. Patients with missing mGPS data were excluded from multivariable analyses, and no imputation was performed. Variables were analyzed as continuous parameters after logarithmic transformation using the natural logarithm (ln) to reduce the effect of skewed distributions. Variables for multivariable analysis were selected based on clinical relevance, univariate significance, and consideration of biological overlap among inflammatory indices. To reduce the risk of overfitting, the number of variables included in the model was limited, and closely related composite markers were not included simultaneously to avoid redundancy. Variance inflation factors (VIFs) were calculated to assess potential multicollinearity among variables included in the multivariate model. Correlation analyses were performed to evaluate relationships between inflammatory indices. Model selection was additionally supported by likelihood-based comparisons, including −2 log likelihood and Akaike Information Criterion (AIC). The final multivariate model included ECOG performance status, histologic subtype, logNLR, and mGPS. Given the overlap among CRP-based inflammatory indices, IBI was evaluated in a separate exploratory model rather than being included simultaneously in the primary model. Hazard ratios (HRs) with 95% confidence intervals (CIs) were calculated. All statistical tests were two-sided, and a p value <0.05 was considered statistically significant. Part of this dataset was previously presented in preliminary form as a conference abstract (ESMO 2025). The current study represents a substantially expanded and methodologically refined analysis of the same cohort.

## 3 Results

### 3.1 Demographic and clinicopathologic characteristics of patients

**Figure 1:**
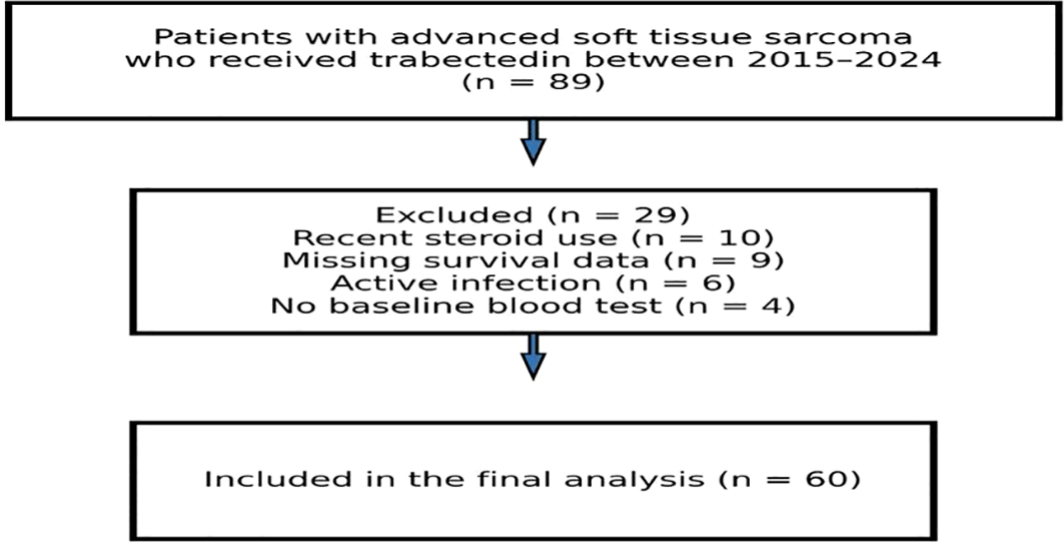
Flow diagram of the study population.

Of the 89 patients evaluated for eligibility, 60 fulfilled the study criteria and were included in the final analysis (**Figure 1****)**.

The median age at the initiation of trabectedin was 54.5 years (range, 35–77), and 37 patients (61.7%) were female. Fourteen patients (23.3%) were aged 65 years or older. Most patients had an ECOG performance status of 0 (58.3%). Leiomyosarcoma was the most common histological subtype, accounting for 55% of cases. At the time of diagnosis, 26 patients (43.3%) had *de novo* metastatic disease. The most frequent primary tumor site was the retroperitoneum (46.7%). Visceral metastases were present in 49 patients (81.7%), most commonly involving the lungs (68.3%). Trabectedin was administered at a median treatment line of three (range, 2–5), and 73.3% of the patients received the drug in the third line or beyond.

**Figure 2:**
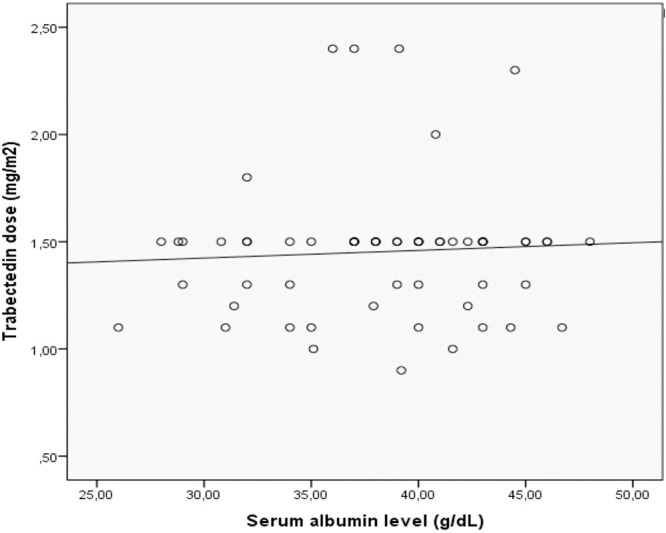
Scatter plot showing the relationship between serum albumin levels and trabectedin dose. A fitted regression line is shown. No significant correlation was observed (Spearman r = 0.045, p = 0.73).

Baseline demographic and clinicopathologic characteristics are summarized in **Table 1**. The median administered dose was 1.5 mg/m² (range, 0.9–2.4), and the median number of treatment cycles was 4 (range, 1–20). Serum albumin level was 3.9 g/dL (median; range, 2.6–4.8). No significant correlation was observed between serum albumin levels and trabectedin dose (Spearman r = 0.045, p = 0.73; **Figure 2**).

**Table 1:** Baseline demographic and clinicopathologic findings for the whole cohort.

Variables			n= 60
**Age**	Median (range)		54.5 (35-77)
	≥ 65 years, n (%)		14 (23.3)
**Gender, n (%)**	Female		37 (61.7)
	Male		23 (38.3)
**ECOG PS, n (%)**	PS 0		35 (58.3)
	PS ≥ 1		25 (41.7)
***De novo* metastatic disease, n (%)**			26 (43.3)
**Pathologic subtype** **n (%)**	Leiomyosarcoma		33(55)
	Liposarcoma	Well differentiated	7 (11.7)
		Dedifferentiated	5 (8.3)
		Myxoid	5 (8.3)
	Other		10 (16.7)
**Primary tumor site** **n (%)**	Retroperitoneum		28 (46.7)
	Trunk		2 (3.3)
	Lower Extremity		11 (18.3)
	Other		7 (11.7)
	Uterine		12 (20)
**Visceral metastases, n (%)**			49 (81.7)
**Lung metastases, n (%)**			41(68.3)
**Liver metastases, n (%)**			17 (28.3)
**Trabectedin treatment line, n (%)**	Median (range)		3 (2-5)
	≥3.line, n (%)		44 (73.3)
**Trabectedin dose, mg/m²**	Median (range)		1.5 (0.9-max 2.4)
	≥ 1.5, n (%)		39 (65)
**Number of trabectedin cycles**	Median (range)		4 (1-20)
	≥ 4, n (%)		37 (61.6)
**Serum albumin level (** g/dL **) (median) (range)**			3.9 (2.6-4.8)
**Modified Glasgow Prognostic Score (mGPS)** n=54 (%)*	mGPS 0		19 (35.1)
	mGPS 1		24 (44.4)
	mGPS 2		11 (20.5)
**Neutrophil/Lymphocyte ratio (NLR) (median) (range)**			2.84 (0.49-12.53)
**Platelet/Lymphocyte Ratio (PLR) (median) (range)**			184.9 (28.5- 474.1)
**Prognostic Nutritional Index (PNI) (median) (range)**			39.6 (26.4- 49.15)
**Pan Immune Inflammation Value (PIV) (median) (range)**			364.9 (20.8-2637.5)
**Inflammatory burden index (IBI) (median) (range)**			49.2 (1.1-4123.4)

*Percentages are calculated based on available data.

### 3.2 Nutritional and inflammation-based indices

According to the mGPS, 19 patients (35.8%) were classified as mGPS 0, 24 (43.3%) as mGPS 1, and 11 (20.9%) as mGPS 2. The median values of the inflammatory and nutritional indices were PNI 39.6 (range, 26.4–49.15), NLR 2.84 (range, 0.49–12.53), PLR 184.9 (range, 28.5–474.1), PIV 364.9 (range, 20.8–2637.5), and IBI 49.2 (range, 1.1–4123.4) (**Table 1**).

### 3.3 Treatment response and survival outcomes

#### 3.3.1 Treatment response rate

Response evaluation was available for 50 patients, as 10 patients were not evaluable due to the absence of radiological assessment. No complete response was observed. Partial response was achieved in 12 patients (24%), stable disease in 16 (32%), and progressive disease in 22 (44%). The objective response rate was 24%, and the disease control rate was 56%.

#### 3.3.2 Survival outcomes

**Figure 3:**
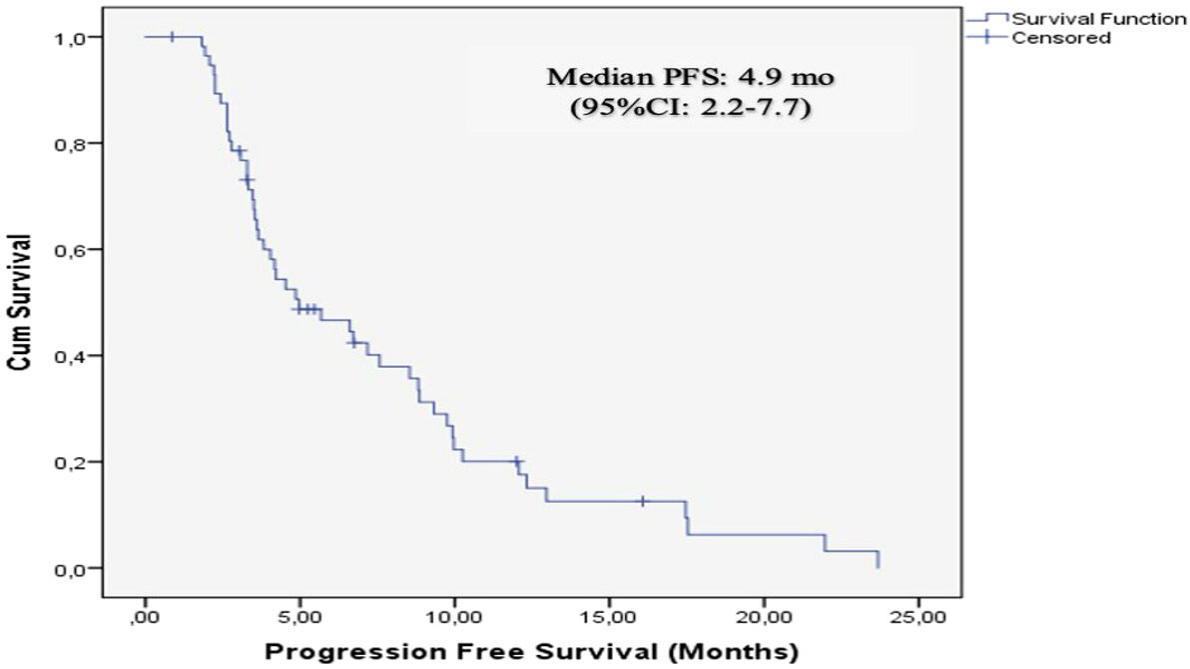
Kaplan-Meier curves for progression- free survival in the entire cohort.

**Figure 4:**
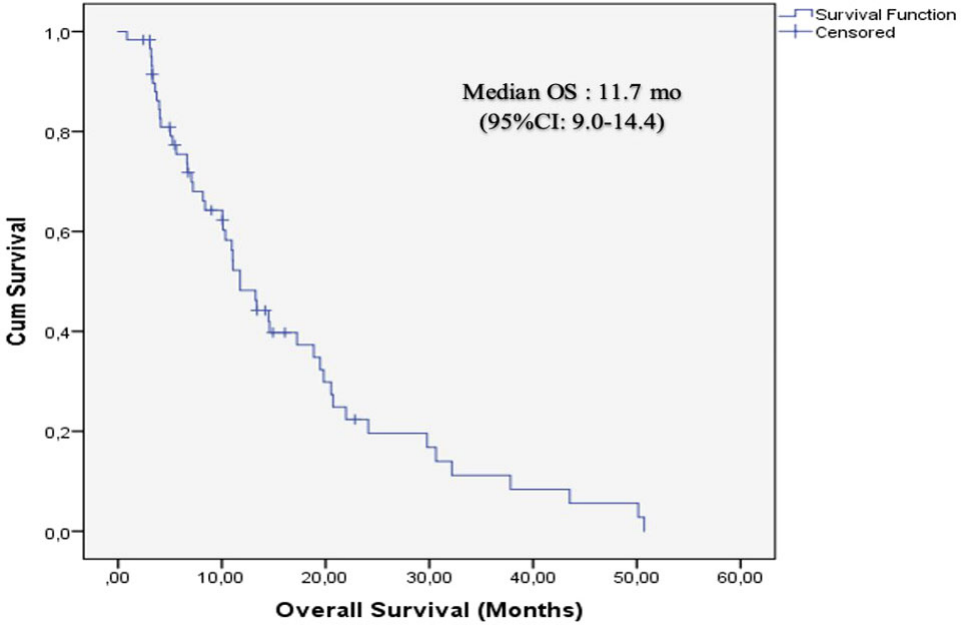
Kaplan-Meier curve for overall survival in the entire cohort.

The data cutoff date for follow-up was May 2025. The median follow-up duration was 10.2 months (range: 1–50.7). During the follow-up, 51 patients (85%) experienced disease progression, and 47 patients (78.3%) died. In the entire cohort, median PFS was 4.9 months (95% CI: 2.2-7.7), and median OS was 11.7 months (95% CI: 9.0–14.4). The 12- and 24-month OS rates were 48% and 16%, respectively (**Figures 3**, **4**). Treatment response and survival outcomes are summarized in **Table 2**.

**Table 2:** Treatment response and survival outcomes.

Variable		n (%)
Treatment Response (n=50) *	Complete Response (CR)	0
	Partial Response (PR)	12 (24)
	Stable Disease (SD)	16 (32)
	Progressive Disease (PD)	22 (44)
	Objective Response Rate (ORR) (%)	24
	Disease Control Rate (DCR) (%)	56
Progression		51 (85)
Progression Free Survival	Median (months)	4.9 (95%CI: 2.2-7.7)
	6. months PFS rate	46 %
	12. months OS rate	20 %
Death		47 (78.3)
Overall Survival	Months (mo)	11.7 (95 % CI: 9.0-14.4)
	12. months OS rate	48%
	24. months OS rate	16%

*Radiologic evaluation could not be performed in ten patients (16.7%).

#### 3.3.3 Cox regression analysis for overall survival

In univariate analysis, histologic subtype, logNLR, logIBI, and mGPS were significantly associated with overall survival. Serum albumin analyzed as a continuous variable showed a trend toward improved overall survival (HR 0.94; 95% CI 0.88–1.00; p = 0.07). In the multivariate Cox regression model including ECOG performance status, histologic subtype, logNLR, and mGPS, performed using a complete-case approach (n = 54), only mGPS remained an independent prognostic factor for overall survival (HR 3.9; 95% CI 1.5–10.0; p = 0.004). Collinearity diagnostics showed low variance inflation factor values for all variables (ECOG PS: 1.30; histologic subtype: 1.09; logNLR: 1.38; mGPS: 1.12), with no evidence of multicollinearity. In addition, no significant correlation was observed between logNLR and mGPS (r = 0.13, p = 0.31). ECOG performance status showed a trend toward significance (HR 0.45; 95% CI 0.1–1.0; p = 0.06), while histologic subtype demonstrated a borderline association with overall survival (HR 2.0; 95% CI 0.9–4.6; p = 0.07). In an alternative multivariate model including IBI instead of mGPS, IBI showed borderline statistical significance after adjustment for age, ECOG performance status, and histologic subtype (HR 1.002; 95% CI 1.00–1.004; p = 0.049). The model including mGPS demonstrated a better fit compared to the alternative model including IBI (−2 log likelihood: 174.10 vs. 179.90; AIC: 182.10 vs. 187.90). In addition, a strong correlation was observed between IBI and mGPS (r = 0.63, p < 0.001), supporting the presence of substantial overlap between these indices. The results of the Cox regression analysis are summarized in **Table 3**.

**Table 3:** Univariate and multivariate Cox regression analysis for overall survival.

Variable		Category	Univariate analysis		Multivariate analysis	
			HR (95%CI)	p	HR (95%CI)	p
Gender		Female	1.1 (0.6-2.1)	0.60		
		Male				
Age		< 65	0.59 (0.20-1.2)	0.15		
		≥ 65				
ECOG PS		PS 0	0.6 (0.3-1.2)	0.17	0.45 (0.1-1.0)	0.06
		PS ≥1				
Pathologic subtype		Leiomyosarcoma	2.2 (1.1-4.5)	**0.02**	2.0 (0.9-4.6)	0.07
		Liposarcoma				
Primary tumor site		Retroperitoneum	1.03 (0.5-1.8)	0.91		
		Other				
Disease Status		*De novo*	1.1 (0.6-2.0)	0.62		
		Recurrence				
Visceral metastasis		Present	0.6 (0.3-1.4)	0.33		
		Absent				
Lung metastasis		Present	0.7 (0.3-1.4)	0.73		
		Absent				
Liver metastasis		Present	1.4 (0.6-2.8)	0.34		
		Absent				
Trabectedin treatment line		≥ 3	0.9 (0.4-1.7)	0.79		
		<3				
Trabectedin dose (continuous) (mg/m^2^)			1.63 (0.53–5.01)	0.38		
Albumin (continuous) ( g/dL)			0.94 (0.88–1.00)	0.07		
logNLR (continuous)			1.1 (1.007 -1.26)	**0.03**	1.0 (0.90-1.3)	0.35
logPLR (continuous)			1.0 (0.9-1.0)	0.12		
logPIV(continuous)			1.0 (1.0-1.0)	0.21		
logIBI(continuous)			1.001(1.0-1.002)	**0.002**		
logPNI (continuous)			0.94 (0.8-1.0)	0.09		
mGPS	mGPS 0		2.8 (1.3-6.1)	**0.006**	3.9 (1.5-10.0)	**0.004**
	mGPS 1-2					

Univariate analyses were performed in the full cohort (n = 60), whereas multivariate analysis was conducted using a complete-case approach including only patients with available mGPS data (n = 54). No imputation was performed for missing data. Continuous variables were analyzed using Cox proportional hazards regression. Bold values indicate statistically significant results (p < 0.05).

**Figure 5:**
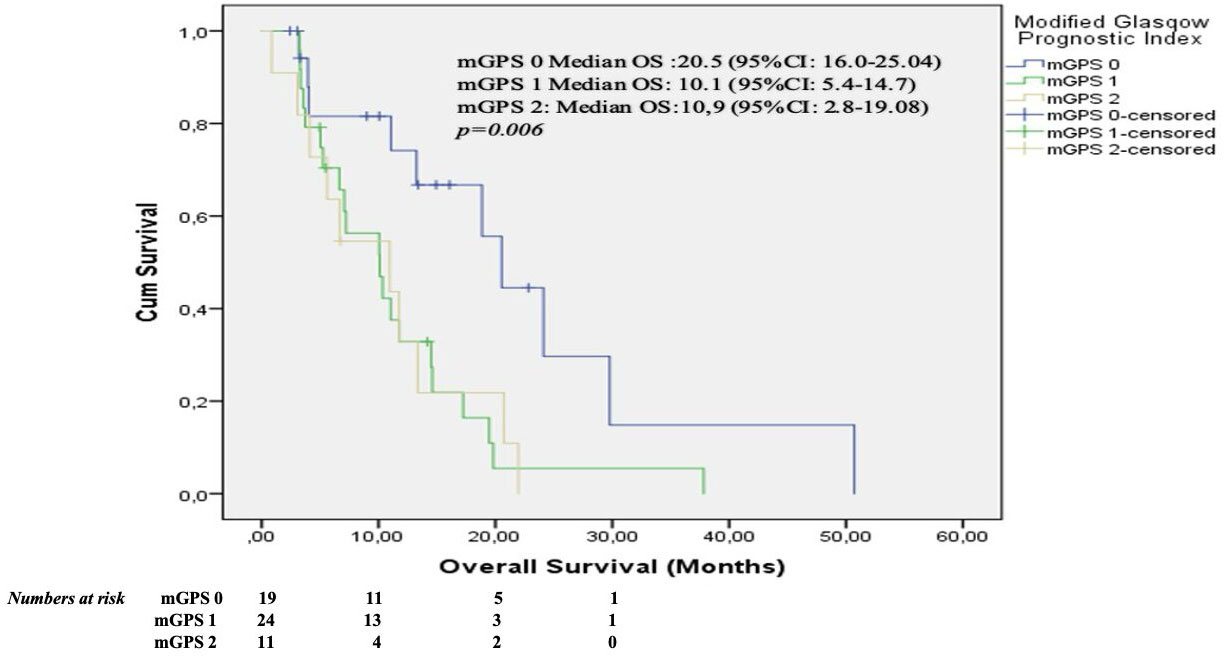
Kaplan-Meier curves for overall survival according to the modified glasgow prognostic score (mGPS). Numbers at risk are shown below.

According to the mGPS categories, the median overall survival was 20.5 months (95% CI 16.0–25.0) for mGPS 0, 10.1 months (95% CI 5.4–14.7) for mGPS 1, and 10.9 months (95% CI 2.8–19.1) for mGPS 2 (p = 0.006) (**Figure 5****).**

## 4 Discussion

This retrospective study analyzed 60 patients with advanced soft tissue sarcoma treated with trabectedin between 2015 and 2024. The median follow-up was 10.2 months, and the median age was 54.5 years. Leiomyosarcoma was the most common subtype (55%), and trabectedin was usually given as third-line therapy. The objective response rate was 24%, and the disease control rate was 56%. Median PFS and OS were 4.9 and 11.7 months, respectively. Patients with a modified Glasgow Prognostic Score (mGPS) of 0 had significantly longer OS compared to those with mGPS 1 or 2 (median OS: 20.5 vs. 10.1 and 10.9 months, respectively; p=0.006), and higher mGPS (1–2 vs 0) remained independently associated with worse overall survival (HR 3.9; 95% CI 1.5–10.0; p = 0.004). These findings provide an overview of the clinical characteristics and outcomes in our cohort and form the basis for the subsequent discussion of prognostic markers.

The clinical outcomes observed in our cohort were consistent with previous data on trabectedin in advanced soft tissue sarcomas. In a phase III trial, trabectedin achieved a median PFS of 4.2 months and a median OS of 12.4 months in patients with previously treated leiomyosarcoma or liposarcoma (5). Likewise, a study conducted by the French Sarcoma Group reported a median PFS of 4.4 months and an OS of 12.2 months (16), which were comparable to those observed in our study (median PFS: 4.9 months; median OS: 11.7 months). In our study, the objective response rate (20%) was numerically higher than those reported in large clinical and real-world analyses. In the RetrospectYon study including 885 patients, the ORR and DCR were 16.5% and 67%, respectively, whereas in the worldwide expanded access program (EAP) involving 1895 patients, the ORR and DCR were 5.9% and 48.5%, respectively (16, 17). Compared with these cohorts, our findings demonstrated a higher ORR and a disease control rate within the reported range. The higher ORR observed in our study may be explained by the retrospective nature of response assessment, potential selection bias, and differences in patient monitoring compared with large multicenter analyses.

Systemic inflammation is well recognized as a key factor influencing cancer development, progression, and resistance to treatment (18, 19). Several inflammation-based indices, including NLR, PLR, and IBI, reflect the balance between tumor-promoting inflammatory processes and host immune response (20, 21). In previous studies of patients with soft tissue sarcoma, elevated NLR and related inflammatory markers have been associated with poorer clinical outcomes, although their independent prognostic value has not been consistently demonstrated across different cohorts (22). Similarly, composite indices of systemic inflammation have shown variable prognostic significance in patients treated with trabectedin, suggesting that their impact may depend on patient characteristics and analytical models (23).

Recent studies have also highlighted the importance of molecular and immune profiling in soft tissue sarcomas. Integrative genomic and transcriptomic analyses have demonstrated that treatment response may be influenced by specific genetic alterations and tumor microenvironment characteristics, including cell cycle–related pathways and immune cell infiltration patterns. For instance, copy number alterations such as CDK4 amplification have been associated with cell cycle activation and poorer clinical outcomes, suggesting a potential mechanism of resistance to systemic therapies. In parallel, transcriptional analyses have shown that tumors from responders may exhibit enrichment of immune-related pathways, including interferon signaling and cytokine–receptor interactions, whereas non-responders tend to demonstrate upregulation of proliferation- and metabolism-related pathways such as glycolysis and hypoxia signaling. Moreover, immune profiling studies have indicated that increased infiltration of immune effector cells, particularly natural killer cells, may be associated with improved treatment response, underscoring the role of the tumor microenvironment in modulating therapeutic efficacy (24–26). These findings support the concept that both tumor-intrinsic molecular features and host-related immune factors contribute to treatment outcomes in STS and highlight the potential value of integrating molecular and inflammatory biomarkers in prognostic assessment.

In addition, nutritional status, as reflected by the prognostic nutritional index (PNI), has been associated with survival outcomes in this setting (27). In our study, although logNLR, logIBI, and logPNI were associated with overall survival in univariate analysis, their contribution was not consistently retained after adjustment. This may be related to the overlap among these indices, the limited sample size, and the possibility that mGPS captures a broader aspect of systemic inflammation and nutritional status. The relatively strong correlation between IBI and mGPS further supports the presence of overlapping inflammatory processes, which may limit their independent contribution when included simultaneously in multivariable models. Taken together, these findings suggest that while individual inflammatory and nutritional indices may provide prognostic information, composite scores such as mGPS may offer a more robust and clinically relevant assessment of systemic inflammatory status in this patient population.

The modified Glasgow Prognostic Score is derived from CRP and albumin. According to the original definition, patients with CRP <10 mg/L are assigned a score of 0 regardless of albumin level. Among patients with CRP ≥10 mg/L, a score of 1 is assigned if albumin is ≥3.5 g/dL and 2 if albumin is <3.5 g/dL. Elevated CRP indicates activation of pro-inflammatory cytokines such as interleukin-6 (IL-6) and tumor necrosis factor-α (TNF-α), whereas reduced albumin levels reflect cytokine-mediated hepatic suppression, diminished metabolic reserve, and impaired nutritional-immune competence (28). These interrelated mechanisms contribute to tumor proliferation, angiogenesis, and immune evasion. In addition to mGPS, recent studies in patients treated with trabectedin have evaluated alternative inflammatory indices, including the lymphocyte-to-monocyte ratio and systemic inflammation index, and have demonstrated their prognostic relevance (22, 23). While these indices were not included in the present study, this reflects differences in predefined study variables rather than selective omission. Importantly, despite methodological differences, these findings consistently support the role of systemic inflammation in determining clinical outcomes. In this context, mGPS may offer complementary and clinically practical prognostic information by integrating both inflammatory and nutritional components. In our cohort, mGPS demonstrated the strongest and independent association with overall survival among all evaluated markers. Patients with mGPS 0 experienced significantly longer survival compared with those with higher scores (mGPS 1 or 2), suggesting that a lower inflammatory and better nutritional status may predict improved outcomes under trabectedin therapy. Trabectedin exerts its antitumor effects not only through DNA binding and transcriptional regulation but also via selective depletion of tumor-associated macrophages, leading to a reduction in pro-inflammatory mediators within the tumor microenvironment (6). These immunomodulatory properties may provide a potential biological context. However, the observed association between mGPS and survival should be interpreted with caution, as mGPS reflects systemic inflammatory and nutritional status rather than tumor-specific immune characteristics such as macrophage infiltration or function. Therefore, the relationship between mGPS and clinical outcomes should be considered hypothesis-generating rather than indicative of a direct mechanistic interaction at the tumor microenvironment level. However, given the retrospective and observational nature of the study, no causal inference can be established. In addition, as albumin is a component of mGPS, alternative explanations for its prognostic value should be considered. Hypoalbuminemia may influence drug pharmacokinetics and treatment tolerability, potentially affecting outcomes independent of systemic inflammation (29). Although no correlation was observed between serum albumin levels and trabectedin dose in our cohort, this finding does not exclude potential pharmacokinetic effects of hypoalbuminemia, which were not evaluated in the present study.

Our results are consistent with the growing body of evidence demonstrating that inflammation-based scores such as mGPS possess prognostic value across a wide range of malignancies, including soft tissue sarcoma. In patients with recurrent high-grade glioma, mGPS has been identified as an independent prognostic factor for overall survival following systemic therapy (30). Likewise, McMillan reviewed over 60 studies and confirmed that mGPS is a reproducible inflammation-based prognostic score observed across multiple tumor types, particularly gastrointestinal, pulmonary, and breast cancers (11). In non-small-cell lung cancer, higher mGPS values have been associated with increased treatment-related toxicity and inferior therapeutic outcomes (31). Beyond single-center reports, a comprehensive meta-analysis including 9,839 patients with colorectal cancer provided high-level evidence that both the Glasgow Prognostic Score and its modified form were significantly associated with overall and cancer-specific survival (32). Specifically in soft tissue sarcomas, several studies have evaluated the prognostic role of mGPS and reported consistent findings. Spence et al. demonstrated that patients with an mGPS of 0 had significantly better overall and cancer-specific survival compared with those with higher scores, and mGPS remained an independent prognostic factor in patients undergoing curative-intent surgery (33). Similarly, comparative analyses have suggested that the conventional mGPS provides a clinically meaningful reflection of oncologic outcomes in STS populations (34). Although recent studies have begun to evaluate inflammatory markers in patients treated with trabectedin, the available evidence remains relatively limited and heterogeneous. In this context, our study extends the existing evidence by evaluating the prognostic role of mGPS in a treatment-specific setting.

Taken together, our findings contribute to the growing body of evidence supporting the prognostic value of inflammation-based indices in patients with advanced soft tissue sarcoma treated with trabectedin. This study represents a multicenter, real-world analysis from Türkiye, reflecting outcomes from routine oncology practice. The results suggest that the patient’s systemic inflammatory and nutritional status may influence survival under trabectedin therapy. The use of routinely measured and easily accessible laboratory parameters such as C-reactive protein and albumin provides a simple way to assess prognosis in daily practice. Although the multicenter design and inclusion of real-world data improve the representativeness of our results, several limitations should be noted. The retrospective nature of the study may have introduced bias, and some clinical variables such as corticosteroid use or comorbidities could not be fully accounted for. Laboratory parameters were assessed only at baseline, and dynamic changes during treatment were not captured. Moreover, because inflammatory indices may be affected by non-cancer-related conditions or inter-laboratory variability, residual confounding cannot be excluded.

Finally, the relatively small sample size limits the statistical power to detect weaker associations. Although the number of events per variable in the multivariable model was within an acceptable range, the overall sample size and number of events may still have limited the precision of the estimates, as reflected by the relatively wide confidence intervals observed in some analyses. Therefore, the study may have been underpowered to detect modest but clinically meaningful associations, particularly for variables with borderline statistical significance, such as histologic subtype. In addition, no formal *a priori* or *post hoc* sample size or power calculation was performed, as the study was retrospective and based on the available cohort. This may further limit the ability to draw definitive conclusions regarding weaker effects. Furthermore, the number of variables included in the multivariate model was limited to reduce the risk of overfitting, and correlated inflammatory indices were not included simultaneously. The observed increase in the hazard ratio for mGPS after multivariable adjustment may reflect negative confounding between clinical and inflammatory variables. Additionally, survival estimates for the mGPS 2 subgroup should be interpreted with caution due to the small number of patients and potential instability of Kaplan–Meier estimates at later time points.

While mGPS has been evaluated in heterogeneous STS populations, recent studies have begun to investigate inflammatory markers in patients treated with trabectedin; however, the available evidence remains limited and heterogeneous, and direct comparisons across indices are challenging. Therefore, our findings may offer treatment-specific prognostic insight. In this context, despite these limitations, data collection from several tertiary oncology centers and the consistent trabectedin treatment protocols across sites support the reliability of the findings. Nevertheless, prospective, multicenter studies with larger cohorts are needed to better define the role of inflammation-based prognostic scores in treatment stratification and clinical decision-making in advanced soft tissue sarcoma.

## Data Availability

The raw data supporting the conclusions of this article will be made available by the authors, without undue reservation.

## References

[1] Mastrangelo G Coindre JM Ducimetière F Dei Tos AP Fadda E Blay JY . Incidence of soft tissue sarcoma and beyond: a population-based prospective study in 3 European regions. Cancer. (2012) 118:5339–48. doi: 10.1002/cncr.27555. PMID: 22517534

[2] Sbaraglia M Bellan E Dei Tos AP . The 2020 WHO classification of soft tissue tumours: news and perspectives. Pathologica. (2021) 113:70–84. doi: 10.32074/1591-951x-213. PMID: 33179614 PMC8167394

[3] Judson I Verweij J Gelderblom H Hartmann JT Schöffski P Blay J-Y . Doxorubicin alone versus intensified doxorubicin plus ifosfamide for first-line treatment of advanced or metastatic soft-tissue sarcoma: a randomised controlled phase 3 trial. Lancet Oncol. (2014) 15:415–23. doi: 10.1016/s1470-2045(14)70063-4. PMID: 24618336

[4] Gronchi A Miah AB Dei Tos AP Abecassis N Bajpai J Bauer S . Soft tissue and visceral sarcomas: ESMO-EURACAN-GENTURIS clinical practice guidelines for diagnosis, treatment and follow-up(⋆). Ann Oncol. (2021) 32:1348–65. doi: 10.1016/j.annonc.2021.07.006. PMID: 34303806

[5] Demetri GD von Mehren M Jones RL Hensley ML Schuetze SM Staddon A . Efficacy and safety of trabectedin or dacarbazine for metastatic liposarcoma or leiomyosarcoma after failure of conventional chemotherapy: results of a phase III randomized multicenter clinical trial. J Clin Oncol. (2016) 34:786–93. doi: 10.1200/jco.2015.62.4734. PMID: 26371143 PMC5070559

[6] Germano G Frapolli R Belgiovine C Anselmo A Pesce S Liguori M . Role of macrophage targeting in the antitumor activity of trabectedin. Cancer Cell. (2013) 23:249–62. doi: 10.1016/j.ccr.2013.01.008. PMID: 23410977

[7] Guthrie GJ Charles KA Roxburgh CS Horgan PG McMillan DC Clarke SJ . The systemic inflammation-based neutrophil-lymphocyte ratio: experience in patients with cancer. Crit Rev Oncol Hematol. (2013) 88:218–30. doi: 10.1016/j.critrevonc.2013.03.010. PMID: 23602134

[8] Szkandera J Absenger G Liegl-Atzwanger B Pichler M Stotz M Samonigg H . Elevated preoperative neutrophil/lymphocyte ratio is associated with poor prognosis in soft-tissue sarcoma patients. Br J Cancer. (2013) 108:1677–83. doi: 10.1038/bjc.2013.135. PMID: 23558897 PMC3668478

[9] Que Y Qiu H Li Y Chen Y Xiao W Zhou Z . Preoperative platelet-lymphocyte ratio is superior to neutrophil-lymphocyte ratio as a prognostic factor for soft-tissue sarcoma. BMC Cancer. (2015) 15:648. doi: 10.1186/s12885-015-1654-6. PMID: 26432433 PMC4592563

[10] Forrest LM McMillan DC McArdle CS Angerson WJ Dunlop DJ . Evaluation of cumulative prognostic scores based on the systemic inflammatory response in patients with inoperable non-small-cell lung cancer. Br J Cancer. (2003) 89:1028–30. doi: 10.1038/sj.bjc.6601242. PMID: 12966420 PMC2376960

[11] McMillan DC . The systemic inflammation-based Glasgow prognostic score: a decade of experience in patients with cancer. Cancer Treat Rev. (2013) 39:534–40. doi: 10.1016/j.ctrv.2012.08.003. PMID: 22995477

[12] Proctor MJ Morrison DS Talwar D Balmer SM Fletcher CD O’Reilly DS . A comparison of inflammation-based prognostic scores in patients with cancer. A Glasgow Inflammation Outcome Study. Eur J Cancer. (2011) 47:2633–41. doi: 10.1016/j.ejca.2011.03.028. PMID: 21724383

[13] Onodera T Goseki N Kosaki G . Prognostic nutritional index in gastrointestinal surgery of malnourished cancer patients. Nihon Geka Gakkai Zasshi. (1984) 85:1001–5. 6438478

[14] Xie H Ruan G Ge Y Zhang Q Zhang H Lin S . Inflammatory burden as a prognostic biomarker for cancer. Clin Nutr. (2022) 41:1236–43. doi: 10.1016/j.clnesp.2022.09.366. PMID: 35504166

[15] Fucà G Guarini V Antoniotti C Morano F Moretto R Corallo S . The Pan-Immune-Inflammation Value is a new prognostic biomarker in metastatic colorectal cancer: results from a pooled-analysis of the Valentino and TRIBE first-line trials. Br J Cancer. (2020) 123:403–9. 10.1038/s41416-020-0894-7 PMC7403416 32424148

[16] Le Cesne A Ray-Coquard I Duffaud F Chevreau C Penel N Bui Nguyen B . Trabectedin in patients with advanced soft tissue sarcoma: a retrospective national analysis of the French Sarcoma Group. Eur J Cancer. (2015) 51:742–50. doi: 10.1016/j.ejca.2015.01.006. PMID: 25727882

[17] Samuels BL Chawla S Patel S von Mehren M Hamm J Kaiser PE . Clinical outcomes and safety with trabectedin therapy in patients with advanced soft tissue sarcomas following failure of prior chemotherapy: results of a worldwide expanded access program study. Ann Oncol. (2013) 24:1703–9. doi: 10.1093/annonc/mds659. PMID: 23385197

[18] Diakos CI Charles KA McMillan DC Clarke SJ . Cancer-related inflammation and treatment effectiveness. Lancet Oncol. (2014) 15:e493–503. doi: 10.1016/s1470-2045(14)70263-3. PMID: 25281468

[19] Crusz SM Balkwill FR . Inflammation and cancer: advances and new agents. Nat Rev Clin Oncol. (2015) 12:584–96. doi: 10.1038/nrclinonc.2015.105. PMID: 26122183

[20] Chan JY Zhang Z Chew W Tan GF Lim CL Zhou L . Biological significance and prognostic relevance of peripheral blood neutrophil-to-lymphocyte ratio in soft tissue sarcoma. Sci Rep. (2018) 8:11959. doi: 10.1038/s41598-018-30442-5. PMID: 30097600 PMC6086886

[21] Liu G Ke LC Sun SR . Prognostic value of pretreatment neutrophil-to-lymphocyte ratio in patients with soft tissue sarcoma: a meta-analysis. Med (Baltimore). (2018) 97:e12176. doi: 10.1097/md.0000000000012176. PMID: 30200120 PMC6133428

[22] Fausti V De Vita A Vanni S Ghini V Gurrieri L Riva N . Systemic inflammatory indices in second-line soft tissue sarcoma patients: focus on lymphocyte/monocyte ratio and trabectedin. Cancers (Basel). (2023) 15:1080. doi: 10.3390/cancers15041080. PMID: 36831421 PMC9954182

[23] Sobczuk P Filipowicz P Lamparski L Kosela-Paterczyk H Teterycz P Kozak K . Systemic inflammation index is a predictive and prognostic factor in patients with liposarcoma or leiomyosarcoma treated with trabectedin. Sci Rep. (2025) 15:5247. doi: 10.1038/s41598-025-89977-z. PMID: 39939795 PMC11821830

[24] Hong JY Cho HJ Yun KH Lee YH Kim SH Baek W . Comprehensive molecular characterization of soft tissue sarcoma for prediction of pazopanib-based treatment response. Cancer Res Treat. (2023) 55:671–83. doi: 10.4143/crt.2022.251. PMID: 36164943 PMC10101793

[25] Rodrigues-Santos P Almeida JS Sousa LM Couceiro P Martinho A Rodrigues J . Immune monitoring of trabectedin therapy in refractory soft tissue sarcoma patients - the IMMUNYON study. Front Immunol. (2025) 16:1516793. doi: 10.3389/fimmu.2025.1516793. PMID: 40007535 PMC11850243

[26] Fetisov T Shtompel P Khazanova S Trapeznikova E Menyailo M Ikonnikov A . Molecular-genetic characteristics of soft tissue sarcomas associated with the development of chemotherapy resistance. J Clin Oncol. (2025) 43:11550. doi: 10.1200/jco.2025.43.16_suppl.11550. PMID: 40980778

[27] Sabe H Takenaka S Kakunaga S Tamiya H Wakamatsu T Nakai S . Prognostic nutrition index as a predictive factor for overall survival in trabectedin-treated advanced soft tissue sarcoma. J Orthop Sci. (2025) 30:171–9. doi: 10.1016/j.jos.2024.02.004. PMID: 38467532

[28] Chojkier M . Inhibition of albumin synthesis in chronic diseases: molecular mechanisms. J Clin Gastroenterol. (2005) 39:S143–6. doi: 10.1097/01.mcg.0000155514.17715.39. PMID: 15758650

[29] Idasiak-Piechocka I Lewandowski D Świgut W Kalinowski J Mikosza K Suchowiejski P . Effect of hypoalbuminemia on drug pharmacokinetics. Front Pharmacol. (2025) 16:1546465. doi: 10.3389/fphar.2025.1546465. PMID: 40051558 PMC11882431

[30] Alan O Telli TA Basoğlu T Arikan R Demircan NC Ercelep O . Prognostic value of modified Glasgow prognostic score in recurrent high-grade glial tumors treated with systemic treatment. Clin Neurol Neurosurg. (2020) 196:105976. doi: 10.1016/j.clineuro.2020.105976. PMID: 32531614

[31] Gioulbasanis I Pallis A Vlachostergios PJ Xyrafas A Giannousi Z Perdikouri IE . The Glasgow prognostic score (GPS) predicts toxicity and efficacy in platinum-based treated patients with metastatic lung cancer. Lung Cancer. (2012) 77:383–8. doi: 10.1016/j.lungcan.2012.04.008. PMID: 22551892

[32] Lu X Guo W Xu W Zhang X Shi Z Zheng L . Prognostic value of the Glasgow prognostic score in colorectal cancer: a meta-analysis of 9,839 patients. Cancer Manag Res. (2019) 11:229–49. doi: 10.2147/cmar.s185350. PMID: 30636896 PMC6307678

[33] Spence S Doonan J Farhan-Alanie OM Chan CD Tong D Cho HS . Does the modified Glasgow prognostic score aid in the management of patients undergoing surgery for a soft-tissue sarcoma?: an international multicentre study. Bone Joint J. (2022) 104-b:168–76. doi: 10.1302/0301-620x.104b1.bjj-2021-0874.r1. PMID: 34969280

[34] Nakamura T Asanuma K Hagi T Sudo A . Modified Glasgow prognostic score is better for predicting oncological outcome in patients with soft tissue sarcoma, compared to high-sensitivity modified Glasgow prognostic score. J Inflammation Res. (2022) 15:3891–9. doi: 10.2147/jir.s369993. PMID: 35845092 PMC9285857

